# *Campylobacter* Infection as a Trigger for Guillain-Barré Syndrome in Egypt

**DOI:** 10.1371/journal.pone.0003674

**Published:** 2008-11-12

**Authors:** Thomas F. Wierzba, Ibrahim Adib Abdel-Messih, Bayoumi Gharib, Shahida Baqar, Amina Hendaui, Ibrahim Khalil, Tarek A. Omar, Hamed E. Khayat, Shannon D. Putnam, John W. Sanders, Lai-King Ng, Lawrence J. Price, Daniel A. Scott, Robert R. Frenck

**Affiliations:** 1 US Naval Medical Research Unit No. 3, Cairo, Egypt; 2 Neurology Department, Alexandria University Hospitals, Alexandria, Egypt; 3 Naval Medical Research Center, Silver Spring, Maryland, United States of America; 4 Neurology Department, Cairo University Hospitals, Cairo, Egypt; 5 Neurology Department, Ain Shams University Children's Hospital, Ain Shams, Egypt; 6 US Naval Medical Research Unit No. 3, Cairo, Egypt; 7 Bacteriology and Enteric Diseases Program, National Microbiology Laboratory, Winnipeg, Manitoba, Canada; 8 Naval Medical Research Center, Bethesda, Maryland, United States of America; Centre de Recherche Public-Santé, Luxembourg

## Abstract

**Background:**

Most studies of *Campylobacter* infection triggering Guillain-Barré Syndrome (GBS) are conducted in western nations were *Campylobacter* infection and immunity is relatively rare. In this study, we explored *Campylobacter* infections, *Campylobacter* serotypes, autoantibodies to gangliosides, and GBS in Egypt, a country where *Campylobacter* exposure is common.

**Methods:**

GBS cases (n = 133) were compared to age- and hospital-matched patient controls (n = 374). A nerve conduction study was performed on cases and a clinical history, serum sample, and stool specimen obtained for all subjects.

**Results:**

Most (63.3%) cases were demyelinating type; median age four years. Cases were more likely than controls to have diarrhea (29.5% vs. 22.5%, Adjusted Odds Ratio (ORa) = 1.69, P = 0.03), to have higher geometric mean IgM anti-*Campylobacter* antibody titers (8.18 vs. 7.25 P<0.001), and to produce antiganglioside antibodies (e.g., anti-Gd1a, 35.3 vs. 11.5, ORa = 4.39, P<0.0001). Of 26 Penner:Lior *Campylobacter* serotypes isolated, only one (41:27, *C. jejuni*, P = 0.02) was associated with GBS.

**Conclusions:**

Unlike results from western nations, data suggested that GBS cases were primarily in the young and cases and many controls had a history of infection to a variety of *Campylobacter* serotypes. Still, the higher rates of diarrhea and greater antibody production against *Campylobacter* and gangliosides in GBS patients were consistent with findings from western countries.

## Introduction

*Campylobacter*–associated diarrhea is common in developing countries and residents experience repeated attacks [1]. In Egypt, for example, *Campylobacter* is the second leading cause of pediatric diarrhea with infants and one year olds experiencing 1.2 and 0.4 episodes per year, respectively [2]. Although most *Campylobacter*-associated diarrhea is self-limited, complications can occur. One complication is Guillain-Barré Syndrome (GBS), an acute, symmetric, ascending paralysis that is estimated to occur 30 times for every 100 000 *Campylobacter* cases. The case fatality ratio approaches 10% [3]–[5].

The link between GBS and campylobacterosis is based on studies suggesting that these enterobacteria are more often isolated from GBS cases than controls along with findings that anti-*Campylobacter* serum antibodies occur more frequently in cases [6]. Studies which have linked *Campylobacter* infection to GBS have been typically performed in developed countries where exposure to *Campylobacter* is rare and residents are likely immunologically naive to *Campylobacter* [1], [6]. Studies examining an association between *Campylobacter* infection and GBS are infrequently performed in the developing world, where in contrast to developed countries, infections with *Campylobacter* are common and residents are repeatedly exposed. In the current study, we examined *Campylobacter* as an agent for GBS in Egypt, a country endemic for campylobacterosis and compared these findings to those reported from developed countries.

## Methods

### Study Population

All patients admitted to Cairo and Alexandria University Hospitals and children admitted to Ain Shams Children's Hospital between April 2001 and September 2003 with GBS or Miller-Fisher syndrome were eligible for enrollment. For GBS, each patient demonstrated a progressive, symmetric ascending paralysis with a relative sensory sparing in more than one extremity with hypo- or areflexia [7]. For Miller-Fisher syndrome, a variant of GBS, patients demonstrated ophthalmoplegia, ataxia and areflexia [8]. If a lumbar puncture was performed, cerebral spinal fluid was evaluated for protein and cell counts. Findings consistent with GBS included an elevated CSF protein (>0.55 g/liter) with a normal CSF cell count (<10 cells/mm3). A neurologist diagnosed each case.

For each case, the next three consecutive age- and hospital-matched patients meeting selection criteria were eligible as controls. Controls were within two years of the case's age, were admitted with an acute illness, and could not present with acute neuropathic symptoms. As we were estimating the frequency of diarrhea before an acute illness, controls like cases could have a history of diarrhea or present with diarrhea but could not have diarrhea as their primary reason for admission. As blood samples from cases were obtained before receiving plasmapheresis or intravenous immunoglobulin, similarly controls were excluded if they received blood or blood products up to 12 months before enrollment.

Clinical data, blood, and three rectal swabs were collected from all subjects. For children and patients too ill to provide a medical history, the history was taken from a parent, spouse, or another adult family member. Nerve conduction studies were performed on each case.

The study was approved by the Institutional Review Board of the US Naval Medical Research Unit No. 3 and the Egyptian Ministry of Health and Population. Voluntary written informed consent for participation was provided by a parent or another adult family member for all cases and controls less than 18 years of age and by the patient if the age was greater than or equal to 18 years, the age of majority. If an adult patient was unable to provide consent due to severe illness, a spouse or another adult family member was consented on behalf of the patient.

### Electrophysiological Data

A Nerve Conduction Velocity (NCV) test was performed using conventional techniques and each patient was categorized as primary demyelinating, primary axonal, inexcitable, equivocal, or normal using published criteria [9]. One investigator (IK) standardized testing at each hospital.

### Microbiologic Investigations

*Campylobacter* isolation was based on techniques suggested elsewhere [10]. Three rectal swabs were collected from each patient. Two swabs were placed in Cary-Blair transport media and one swab in Campy-Thio-Broth. Specimens were refrigerated at 4° to 6°C and transported within three days to the US Naval Medical Research Unit No. 3 (NAMRU-3) laboratory in Cairo.

At NAMRU-3, swabs from the Cary-Blair transport tubes were streaked on *Campylobacter* Selective Agar with Preston antibiotic supplement (CA), charcoal cefoperazone deoxycholate agar (CCDA), Shigella-Salmonella Agar (SS), and MacConkey's Agar (MAC) plates. *Campylobacter*-Thio tubes were incubated at 42°C for two hours then inoculated on *Campylobacter* selective agar plates. The CA and CCDA plates were incubated at 42°C under microaerophilic conditions for up to 72 hours and checked every 24 hours for growth. A case was *Campylobacter* negative, if no growth was found after 72 hours.

When *Campylobacter*-like colonies were observed on the CA and/or CCDA plates, an oxidase test and Gram stain were performed followed by re-streaking onto blood agar plates. After 24 hours of microaerophilic growth, isolates were tested for hippurate hydrolysis. Hippurate positive colonies (presumptive identification for *C jejuni*) were sub-cultured onto three Mueller-Hinton blood agar plates with filters, incubated at 42°C for 24 to 48 hours under microaerophilic conditions. Indoxyl Acetate Hydrolysis (IAH) was conducted on hippurate-negative colonies followed by catalase testing if the IAH was positive. IAH/catalase positive organisms were classified as *C coli* whereas IAH positive/catalase negative colonies were classified as *C upsaliensis*. Multiple *Campylobacter* isolates from a pure plate were pooled and inoculated into three, 1.5 mls of trypticase soy broth supplemented with 15% sterile glycerol and frozen at −70°C.

### Serum Immune Responses

Blood samples were collected for cases within one day of admission (Interquartile Range (IQR): 0 to 2) and for controls on the day (IQR: 0 to 1) of enrollment. Serum was separated and stored at −70°C until assayed by enzyme linked immunoassay (ELISA) described in detail elsewhere [11], [12]. In brief, a glycine extract (GE) of proteins [13] from a reference strain, *C jejuni* 81–176, was prepared. The extract was used to coat wells of MaxiSorp 96-well immuoplates (NUNC) at 3 µg/ml or, for control wells, at 10 µg/ml of bovine serum albumin (Sigma Chemicals). Serum IgM and IgG were measured by ELISA that used peroxidase-conjugated isotype-specific goat anti-human immunoglobulin. Endpoint titers are expressed as the reciprocal of the highest dilution giving a net absorbance (antigen well – BSA control well) value of 0.15 at OD_405_.

### Antiganglioside IgG Responses

Antiganglioside serum IgG was determined by ELISA with minor modification of methodology reported by Willison et al [14]. Briefly, MaxiSorp 96-well immuoplates were coated with 0.5 µg of each ganglioside GM1, GM2, GD1a, Gd1b or Gt1b (Sigma Chemicals). Negative control wells were coated with methanol. After blocking with 1% BSA, plates were washed with PBS-T (PBS with .05% Tween_20_) and 100 µl of 1∶100 diluted samples were added in duplicate to each ganglioside and control wells. Following overnight incubation at 4°C, plates were washed 6 times and 100 µl of 0.1% BSA containing 0.25 µg of HRP conjugated goat anti-human IgG (KPL) was added and incubated for two hours at 4°C. Following washing, 100 µl of substrate ABTS+H_2_O_2_ (KPL) was added to each well and after an additional 30 minutes incubation at room temperature, absorbance (OD_405_) was measured (Molecular Devices). Net OD was calculated by subtracting the OD values of control well from the ganglioside-coated wells. Data are expressed as net OD_405_ at 1∶100 serum dilutions.

### *Campylobacter* Speciation and Serotyping

Biochemical speciation of *Campylobacter* isolates was confirmed using a two-step Polymerase Chain Reaction (PCR) based test [15]. A region in the 16S rRNA was amplified and digested with the endonucleases *DdeI*, *BsrI*, and *TaqI*. Resulting PCR products were separated by gel electrophoresis, stained with ethidium bromide and visualized by UV. Patterns were then evaluated by comparison to reference standards. Identification of *C jejuni* or *C coli* was confirmed by amplification of the hippurate gene (hipp) or the aspartyl kinase gene (asp). All *Campylobacter* isolates were serotyped using the methods of Penner and Lior [16], [17].

### Definitions

Subjects were thought to possess luxury items if they owned three or more of the following: washing machine, sewing machine, oven, apartment, farmland, other land, or cell phone. Participants owned livestock if they kept goats, cows, ducks, pigeons, etc. Patients produced antiganglioside antibodies if the net OD (gangliosides coated wells – control wells) readings was >.01, the detectable limit of the assay, while those not producing antiganglioside antibodies were patients with an OD of ≤0.1. To be consistent with a previous study, a diarrhea case was a patient with a history of diarrhea in the three months preceding admission [18]. *Campylobacter*-associated diarrhea was defined as isolating *Campylobacter* from a patient reporting diarrhea.

### Statistical Analysis

A conditional multivariate logistic regression model that accounts for the lack of sampling independence from matching was used to compare cases to controls for demographic characteristics, diarrhea prevalence, etc. [19]. A univariate odds ratio was obtained by adding a single dependent (i.e., case or controls status) and independent variable to the model. When controlling for confounders, additional independent variables were added including, unless stated otherwise, gender, livestock ownership, and possession of luxury items.

When doing a subgroup analysis of cases and controls (e.g., cases and control with diarrhea) matching by age and hospital was incomplete. In this situation, we employed a multivariate unconditional regression and fit age and hospital of admission. Because age was not normally distributed, this variable was fit as a categorical variable: <5 year old (infants and toddlers), 5 to <13 years (children), 13 to <18 (teenagers), and 18 or older (adults). For all models, the 95% confidence intervals and p-values were obtained from the model parameters.

When comparing cases to controls by IgM serum antibody titers, we used linear regression with Generalized Estimating Equations because of matching [20], [21]. Univariate models included a single dependent (i.e., normal log of serum IgM titer). For all multivariate models, gender, livestock ownership, and possession of luxury items were included. When completing a subgroup analysis (e.g., only cases and control with diarrhea) age and hospital of admission were added to the model.

When analyzing only case data (e.g., *Campylobacter* excretion), an unconditional multivariate logistic regression was used. The values for univariate and multivariate models were calculated as the conditional regression model described earlier.

The distribution of *Campylobacter* species and serotypes for cases and controls were compared using a Fisher's exact test since cell sizes were small (i.e., <5). A sign rank test was employed to compare the distribution of ages between cases and controls expressing *Campylobacter* and the distribution of days from admission to enrollment. To determine if diarrhea was more common during warmer months and whether males were more likely to have GBS than females, the exact p-value of a binomial test of significance with binomial probability under the null hypothesis of 0.5 was used [22]. A chi-square test was employed to compare the clinical characteristics (e.g., mucoid stools, fever) of cases and controls reporting diarrhea.

Statistical significance was two tailed with P<0.05. For analyses, SAS version 9.1 (SAS Institute, Inc, Cary, North Carolina) or StatXact version 4.01 (Cytel Software Corporation, Cambridge, Massachusetts) was used. For graphing, SigmaPlot version 8.0 (SPPS, Inc, Chicago, Illinois) was employed.

## Results

### Enrollees

Neurologists identified 138 GBS patients of which 133 (96.4%) including one case of Miller-Fisher Syndrome were enrolled (one declined to participate, three had normal NCV, and one was infected with *Shigella flexneria*). Out of 401 matched-controls, 374 (93.3%) were enrolled (eight did not meet inclusion criteria and 19 were infected with *Salmonella* spp.) Rectal swabs and serum samples were obtained from all enrollees.

### Description of Cases

Of the cases, 120 (90.2%) received a NCV test (4 died and 9 left the hospital before testing) with 76 (63.3%) found to have primary demyelinating disease, 17 (14.2%) had primary axonal disease, 15 (12.5%) had inexcitable disease and test results were equivocal for 12 (10.0%) patients.

GBS showed no seasonality (P = .20). GBS was more common in males (60.2%, P = 0.02). Twenty-one (15.8%) cases required mechanical ventilation and 11 (8.3%) cases died.

### Case and Control Characteristics

Cases ranged in age from 7 months to 77 years with a median of 4 years (IQR: 2 to 9) while controls ranged from 1 month to 75 years with a median of 4 years (IQR: 1 to 9). (Table 1) Cases and controls appeared similar for gender and possession of luxury items. Cases were more likely to own livestock (P = 0.002). Most cases and controls were from Alexandria University Hospital (51.1%), followed by Cairo University Hospitals (36.9%), and Ain Shams University Children's Hospital (12.0%). Median age of cases at the admitting hospital was 5 (IQR: 2 to 13), 4 (IQR: 2 to 5), and 2 (IQR: 1 to 6.5) years for Alexandria, Cairo, and Ain Shams Pediatric Hospitals, respectively.

**Table 1:** Characteristics of cases and controls enrolled in study of *Campylobacter* infection and Guillain-Barré Syndrome, the Arab Republic of Egypt, April 2001 through September 2003.

Population characteristics	All Cases and controls		History of diarrhea		*Campylobacter* positive	
	Cases (n = 133)	Controls (n = 374)	Cases (n = 39)	Controls (n = 83)	Cases (n = 14)	Controls (n = 26)
Age (years)^a^^,^ b	4 (2 to 9)	4 (1 to 9)	4 (1 to 19)	3 (1 to 10)	4 (2.5 to 9)	1.5 (1.0 to 6.0)
Male (%)	60.2	57.0	56.4	51.8	78.7	53.9
Own livestock (%)	51.5	35.1^c^	39.5	32.5	42.9	50.0
Possessed luxury items (%)^d^	28.6	23.8	28.2	33.7	7.1	42.3^e^
Ain Shams University, Children's	12.0	12.3	20.5	22.9	14.3	11.5
Cairo University	36.8	35.8	18.0	19.3	50.0	38.5
Alexandria University	51.1	51.9	61.5	57.8	35.7	50.0

a Median (interquartile range).

b Patients were matched to controls for age (±2 years) and hospital for all cases and controls; matching was incomplete when analyzing subgroups (e.g., Cases and controls with diarrhea).

c P = .002; Odds Ratio = 1.86 (95% CI: 1.25 to 2.78), univariate unconditional logistic regression.

d Owned three or more luxury items (e.g., cell phone, car).

e P = .04; Odd Ratio = 0.10 (95% CI: 0.01 to 0.93), univariate unconditional logistic regression.

### Diarrhea Episodes

Thirty-nine (29.5%) cases and 83 (22.5%) controls (1 case and 5 controls had incomplete diarrhea data) reported having diarrhea with cases being 1.69 (95% CI: 1.04 to 2.75, P = 0.03) times more likely to have diarrhea after adjusting for confounding variables. Diarrhea was more common during warmer months but only reached statistical significance for controls, 63.9% (P = 0.015), but not for cases, 61.5% (P = .20). The number of days between diarrhea onset and hospital admission was statistically similar for cases (median = 14, IQR: 2 to 35) and controls (median = 14, IQR: 7 to 28). Among case and controls reporting diarrhea, there were no statistically significant differences for reports of mucoid stools (38.2% vs. 51.9%), bloody stools (14.3% vs. 11.5%), fever (43.2% vs. 58.7%), or vomiting (18.4% vs. 30.1%).

### *Campylobacter* Isolation and Serotypes

*Campylobacter* isolates were identified from 14 (10.5%) cases and 26 (7.0%) controls (OR_a_ = 1.66, 95% CI: 0.78 to 3.51, P = .19). Of the *Campylobacter* isolates, *C jejuni* was isolated from 11 (78.6%) cases and 23 (88.5%) control, while the remaining isolates were *C coli* (P = 0.65 for distribution by species). Six (42.8%) of 14 case isolates and 7 (28.0%) of 25 control isolates (one control had missing data) had diarrhea. We did not detect an association between *Campylobacter* isolation and diarrhea.

There were 26 Penner:Lior serogroups identified. (Table 2) Of these serogroups, 4 (3.0%) case strains and 1 (0.3%) control strain were Penner:Lior 41:27, a *C jejuni* strain (P = 0.02). None of the other 25 serogroups were statistically associated with GBS.

**Table 2:** Percent of each Penner and Lior (Penner:Lior) *Campylobacter* serogroup isolated from Guillain-Barré Syndrome cases and patient-controls, the Arab Republic of Egypt, April 2001 through September 2003.

Penner:Lior Group	Cases^a^ (n = 133)	Controls (n = 374)	P-value^b^
	% (n)	% (n)	
1(10, 32, 47):42_C_ c	0 (0)	0.3 (1)	1.0
1(10, 44, 47):UT_C_	0 (0)	0.3 (1)	1.0
1:UT_J_	0 (0)	0.5 (2)	1.0
2:125_J_	0 (0)	0.3 (1)	1.0
4 (13, 50):7_J_	0 (0)	0.3 (1)	1.0
5 (32):22_J_	0 (0)	0.3 (1)	1.0
5:UT_J_	0 (0)	0.5 (2)	1.0
10(47): 69_J_	0 (0)	0.3 (1)	1.0
13:36_J_	0.8 (1)	0.3 (1)	0.5
13(50, 65):7_J_	0.8 (1)	0 (0)	0.3
14:57_C_	0 (0)	0.3 (1)	1.0
19:UT_J_	0.8 (1)	0 (0)	0.3
19 (33): UT_J_	0.8 (0)	0.3 (1)	1.0
22:UT_J_	0.8 (1)	0.5 (2)	1.0
24:110_C_	0 (0)	0.3 (1)	1.0
32:22_J_	0 (0)	0.3 (1)	1.0
34:46_C_	0.8 (1)	0 (0)	0.3
37(56):28_C_	0 (0)	0.3 (1)	1.0
40:80_J_	0.8 (1)	0 (0)	0.3
41:27_J_	3.0 (4)	0.3 (1)	0.02
42:19_J_	0 (0)	0.5 (2)	1.0
46(47):95_C_	0.8 (1)	0 (0)	0.3
46(47):UT_C_	0 (0)	0.3 (1)	1.0
49:97_C_	0 (0)	0.3 (1)	1.0
53:UT_J_	0 (0)	0.3 (1)	1.0
56:UT_C_	1.5 (2)	0 (0)	0.07

a One case specimen and two controls specimens were not serotyped.

b Fisher's exact test.

c Subscript “J” = *C jejuni* and subscript “C” = *C coli*.

### Serum Antibody Responses

**Figure 1:**
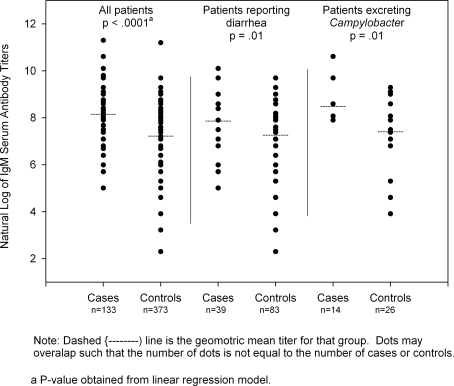
Natural log of IgM serum antibody titers against Campylobacter among Guillain-Barré Syndrome cases and patient-controls and among cases and controls reporting diarrhea or excreting Campylobacter, the Arab Republic of Egypt, April 2001 through September 2003.

We detected significantly higher geometric mean, IgM serum antibody titers in cases than controls (Geometric Mean Titers (GMT): 8.18 vs. 7.25, P<0.0001). (Figure 1) Cases had higher GMT when restricting the analysis to cases and controls reporting diarrhea (GMT: 7.89 vs. 7.29, P = 0.01) or excreting *Campylobacter* (GMT: 8.51 vs. 7.43, P = 0.01). These differences remained statistically significant after adjusting for confounders.

### Antiganglioside Responses

Except for GM2, the proportion of cases producing autoantibodies against gangliosides was statistically greater for cases than controls. (Table 3) The odds of having antiganglioside antibodies for cases was 2.79 (95% CI: 1.77 to 4.40, P<0.0001), 4.39 (95% CI: 2.59 to 7.45, P = 0.047), 2.95 (1.77 to 4.90, P<0.0001), and 2.56 (95% CI: 1.29 to 5.07, P = 0.007) for GM1, GD1a, GD1b, and GT1b, respectively.

**Table 3:** Odds of having IgM serum antibodies against GMI, GM2, GD1a, GD1b, and GT1b gangliosides in Guillain-Barré Syndrome cases and patient-controls for all cases and controls, for patients reporting diarrhea, and for patients excreting *Campylobacter*, the Arab Republic of Egypt, April 2001 through September 2003.

Antiganglioside Antibodies	All cases and controls			History of diarrhea			*Campylobacter* positive		
	Cases (n = 133)	Controls (n = 374)	Adjusted Odds Ratio^a^	Cases (n = 39)	Controls (n = 83)	Adjusted Odds Ratio^b^	Cases (n = 14)	Controls (n = 26)	Adjusted Odds Ratio^c^
GM1	45.9	25.9	2.79 (1.77 to 4.40)^d^^,^ e	35.9	26.5	1.62 (0.70 to 3.76)	50.0	15.4	5.17 (1.03 to 26.07)^f^
GM2	11.3	13.7	0.77 (0.41 to 1.47)	2.6	16.9	0.14 (0.02 to 1.12)	7.1	11.5	0.50 (0.04 to 5.92)
GD1a	35.3	11.5	4.39 (2.59 to 7.45)^e^	28.2	9.6	5.03 (1.67 to 15.18)^g^	42.9	7.7	7.89 (1.16 to 53.65)^h^
GD1b	30.8	13.1	2.95 (1.77 to 4.90)^e^	18.0	15.6	1.37 (0.48 to 3.93)	57.1	7.7	23.84 (2.46 to 231.25)^i^
GT1b	14.3	6.7	2.56 (1.29 to 5.07)^j^	7.7	6.0	1.35 (0.29 to 6.36)	28.6	3.9	6.22 (0.60 to 64.97)

a Conditional logistic regression controlling for age, gender, ownership of farm animals, and ownership of luxury items.

b Unconditional logistic regression controlling for age, gender, livestock ownership, and possession of luxury items.

c Unconditional logistic regression controlling for possession of luxury items. Other variable were excluded because of sparse data.

d Odds Ratio (95% CI).

e <.0001.

f P = 0.047.

g P = .004.

h P = 0.04.

i P = 0.006.

j P = .007.

When limiting the analysis to patients reporting diarrhea, antiganglioside antibody production was again higher in cases than controls except for GM2. However, the difference was only statistically significant for autoantibodies against GD1a (ORa = 5.18, 95% CI: 1.76 to 15.68, P = 0.0037). When further restricting the analysis to cases and controls excreting *Campylobacter*, serum antibodies to gangliosides appeared substantially higher in cases than controls and three of the five ganglioside types were statistically significant. Cases were 5.17 (95% CI: 1.03 to 26.07, P = 0.047), 7.89 (95% CI: 1.16 to 53.65, P = 0.04), and 23.84 (95% CI: 2.46 to 231.25, P = 0.006) times more likely than control to produce autoantibodies against GM1, GD1a, and GD1b, respectively.

## Discussion

GBS is believed to be an autoimmune disease. During infection, antibodies may be produced against lipo-oligosaccharides molecules on the surface of some *Campylobacter* strains that mimic the molecular structure of gangliosides [23]. These autoantibodies are then directed against peripheral nerve tissue leading to myelin inflammation, conduction block, and in some cases, axonal degradation. As not all *Campylobacter* infections lead to GBS, the agent and host factors that induce pathogenesis remain unclear.

This study was implemented in Egypt where residents repeatedly experience *Campylobacter*-associated diarrhea [2], [24], [25]. Since most studies of *Campylobacter* and GBS have been completed in western developed countries, where infection and immunity is uncommon, we had an opportunity to examine the association of *Campylobacter* and GBS were residents have opportunities for repeated exposure to *Campylobacter*.

When limiting our analysis to hospitals serving both children and adults, most GBS cases occurred in toddlers. This observation and observations from other developing countries contrasts [26] reports from developed countries where GBS is primarily an adult illness [1]. The age-specific incidence of campylobacterosis which suggests a higher incidence in children living in developing countries and a higher incidence in adults in developed countries appear to correlate with the age-specific incidence of GBS.

We observed that patients in contact with livestock appeared at increased risk of GBS suggesting that animal husbandry may be a hazard for GBS in Egypt. We have previously demonstrated in Egypt that exposure to farm animals increases the risk of *Campylobacter* infection in children [2], which may link animal husbandry to GBS.

More than one in five controls reported diarrhea and controls had IgM antibodies against *Campylobacter* antigens further supporting the view that *Campylobacter* infection was common. Still, GBS patients were 69% more likely to have diarrhea and had significantly higher levels of serum antibodies against *Campylobacter* and several nerve gangliosides. These results suggest that GBS cases were more likely to be exposed to *Campylobacter*, some of which may have been neruopathogenic strains that express lipo-oligosaccharide mimics [3], [27].

As *Campylobacter* exposure was more common in this setting, we were able to compare immune responses between *Campylobacter*-positive cases and *Campylobacter*-positive controls. Despite currently excreting *Campylobacter*, suggesting current exposure, we detected significantly raised anti-*Campylobacter* antibody titers and anti-ganglioside antibodies in cases. A more vigorous antibody-mediated immunity in cases relative to controls may have been due to a greater proportion of case strains expressing lipopolysaccharide mimics or other antigenic variations [28], cases had a greater genetic susceptibility or other host factors to a virulent infection. However, studies of a genetic predisposition to GBS, such as HLA type, have had limited success [29], [30].

We identified a diversity of Penner:Lior serotypes but did not identify a *Campylobacter* serotype associated with a significant number of GBS cases as has been reported elsewhere [31], [32]. A Penner serotype O:41 (i.e., 41:27), *C jejuni*, was statistically associated with GBS. This serotype has been isolated from GBS cases in South Africa [33], [34] and is reported to have lipopolysaccharides that mimic the GM1 ganglioside [35]. Penner serotypes O:1, O:2, O:4, O:10, and O:37 were recovered, and these strains have been reported to be associated with GBS, but we found these isolates only in control stools [3], [18], [36]–[39]. This observation demonstrates the need for studies with comparison populations.

The severity of GBS in Egypt appears similar to that reported from developing countries [40]. The proportion ventilated, about 16%, and the proportion that died, about 8%, is similar to the 20% ventilation rate and 5% to 10% case fatality ratio suggested for developed countries [5], [41].

Before stating our conclusions, we identify study limitations. We have shown that *Campylobacter* are excreted for about 14 days after infection in Egypt, and it is likely that patient stopped excreting *Campylobacter* before admission [2]. Thus, *Campylobacter* excretion is likely higher in cases than reported here. As we interviewed hospital patients, and they were told the study objectives during informed consent, it may be that recall bias increased the frequency of diarrhea reports. Our conclusions could have been inaccurate due to enrollment or case-ascertainment bias, but all cases of acute flaccid paralysis were evaluated, and we had 96% enrollment. Case ascertainment, clinical evaluation, and study implementation could have differed between hospitals thereby biasing the results. To mitigate these differences, we employed identical training, uniform study specific procedures, and centralized supervision.

In conclusion, *Campylobacter* isolation rates and diarrhea histories in our Egyptian patient population suggested that *Campylobacter*-associated infections are not rare. Still, even with widespread exposure to *Campylobacter*, *Campylobacter* infections appeared associated with induction of antiganglioside antibodies that may have triggered paralysis.
